# Vegetation diversity pattern during spring season in relation to topographic and edaphic variables in sub-tropical zone

**DOI:** 10.1186/s40529-023-00398-5

**Published:** 2023-09-16

**Authors:** Hazrat Ali, Zahir Muhammad, Muhammad Majeed, Robina Aziz, Adam Khan, Wali Muhammad Mangrio, Hazem Ghassan Abdo, Hussein Almohamad, Ahmed Abdullah Al Dughairi

**Affiliations:** 1 Department of Botany, University of Peshawar, Peshawar, Pakistan; 2 Department of Botany, University of Gujrat, Hafiz Hayat Campus, Gujrat, 50700 Punjab Pakistan; 3 Department of Botany, Government College, Women University Sialkot, Sialkot, 51310 Punjab Pakistan; 4 Department of Botany, University of Lakki Marwat, Khyber Pakhtunkhwa, Pakistan; 5 Department of Zoology, Faculty of Natural Sciences, Shah Abdul Latif University, Khairpur, 66111 Sindh Pakistan; 6 Geography Department, Faculty of Arts and Humanities, Tartous University, Tartous, Syria; 7 Department of Geography, College of Arabic Language and Social Studies, Qassim University, Buraydah, 51452 Saudi Arabia

**Keywords:** Vegetation diversity, Diversity indices, Spring season, Topography, Environment

## Abstract

**Background:**

The present study was conducted to explore the diversity pattern of spring vegetation under the influence of topographic and edaphic variables in sub-tropical zone, District Malakand. In the present vegetation study, 252 species of 80 families were recorded in the study area. It included 39 species of trees, 43 species of shrubs, 167 species of herbs and 3 climber species. As a whole, 12 communities were established on the basis of topographic and edaphic characteristics in 12 different stations.

**Results:**

The results of the present study revealed that all diversity indices (species diversity, evenness index, species richness index, maturity index) during spring showed that the communities in plains lying at lower altitudes had higher diversity while the communities formed at high altitudes had lower diversity. The results of the similarity index showed that there was low similarity (below 50%) amongst the communities in different stations.

**Conclusions:**

It can be concluded that variations in topographic and edaphic factors affect species diversity and communities pattern.

## Background

Species distribution modelling (SDM) is among the wide-ranging approaches used by contemporary ecologists, conservationists and forest managers to infer environmental variables (Khan et al. 2022; Correia Filho et al. 2023; Chandra et al. 2023) influencing past, current, or upcoming species distribution pattern. Vegetation is the collective growth of plants combine together in a certain area that is the outcome interaction of many factors like elevation, topography, soil characteristics, species composition and biotic interferences (Ahmad et al. 2014; Mandal and Joshi 2014; Abdo et al. 2022). It is the most significant biotic component as it regulates and maintain the ecosystem. Species diversity expresses community structure, composition and habitat conditions (Haq et al. 2022; Jamil et al. 2022). The distribution of plant species within communities are often regulated by climate or climate-influenced ecological factors (Shaheen et al. 2011a, b). Therefore, diversity tends to increase as the environment becomes more favorable and more predictable (Ahmad et al. 2022; De Bello et al. 2013; Hassan et al. 2022; Ilyas et al. 2020; Khan et al. 2014; Kumar and Sharma 2014; Majeed et al. 2021b; Malik and Husain 2006; Nisar et al. 2014; Pande et al. 2002; Qureshi et al. 2012; Rashid et al. 2018; Shah et al. 2014; Shaheen et al. 2012; Ter Braak 1987; Tiwari and Mishra 2016; Wang et al. 2016). It may be attributed that differences in the altitude, aspect and topography results in the variations in species diversity (Moeslund et al. 2013; Dar and Sundarapandian 2016). Climate is a key factor which strongly affect the vegetation (Ali et al. 2017; van Breugel et al. 2019). Among the climatic factors, altitude is the principal controlling factor in vegetation growth. It is an important factor affecting species composition and structure. Variations in plant species composition along altitude is well establishment phenomenon. Aspecthas great influence on the vegetation density, distribution and diversity (Bocksberger et al. 2016; Khan et al. 2019b). Its prediction is of prime importance for the forest ecosystem conservation and management. Composition and diversity of vegetation is reflecting by the aspect and study of which is of a central theme in vegetation ecology. Topography is the key controlling factor in vegetation growth. Topography is considered to exert influences on the plant distribution at regional and landscape levels (Shaheen et al. 2011a, b; Leonti et al. 2015; Ali et al. 2017). Similarly, edaphic factors have an important role in plant growth and development. Soil properties are found to be remarkably correlated with the formation of plant communities (Malik 2013; Silva et al. 2021). Differences in the soil and topographic factors brings variations in species diversity, richness, evenness and maturity among different ecological communities (Khan et al. 2019a; Abdo 2018; Caballero-Serrano et al. 2019). Several studies (Khan et al. 2017a, b; Rahman et al. 2021) have been conducted on vegetation under topographic and edaphic aspects in different parts of the world. The detailed study objectives include, (1) to describe the diversity pattern of the communities under topographic and edaphic factors in subtropical zone, District Malakand (2)To asses potential distribution of vegetation under current and future climate change scenarios and (3)Identifying the most influential climatic factors influencing the spread of vegetation.

## Materials and methods

### Study site

**Fig. 1:**
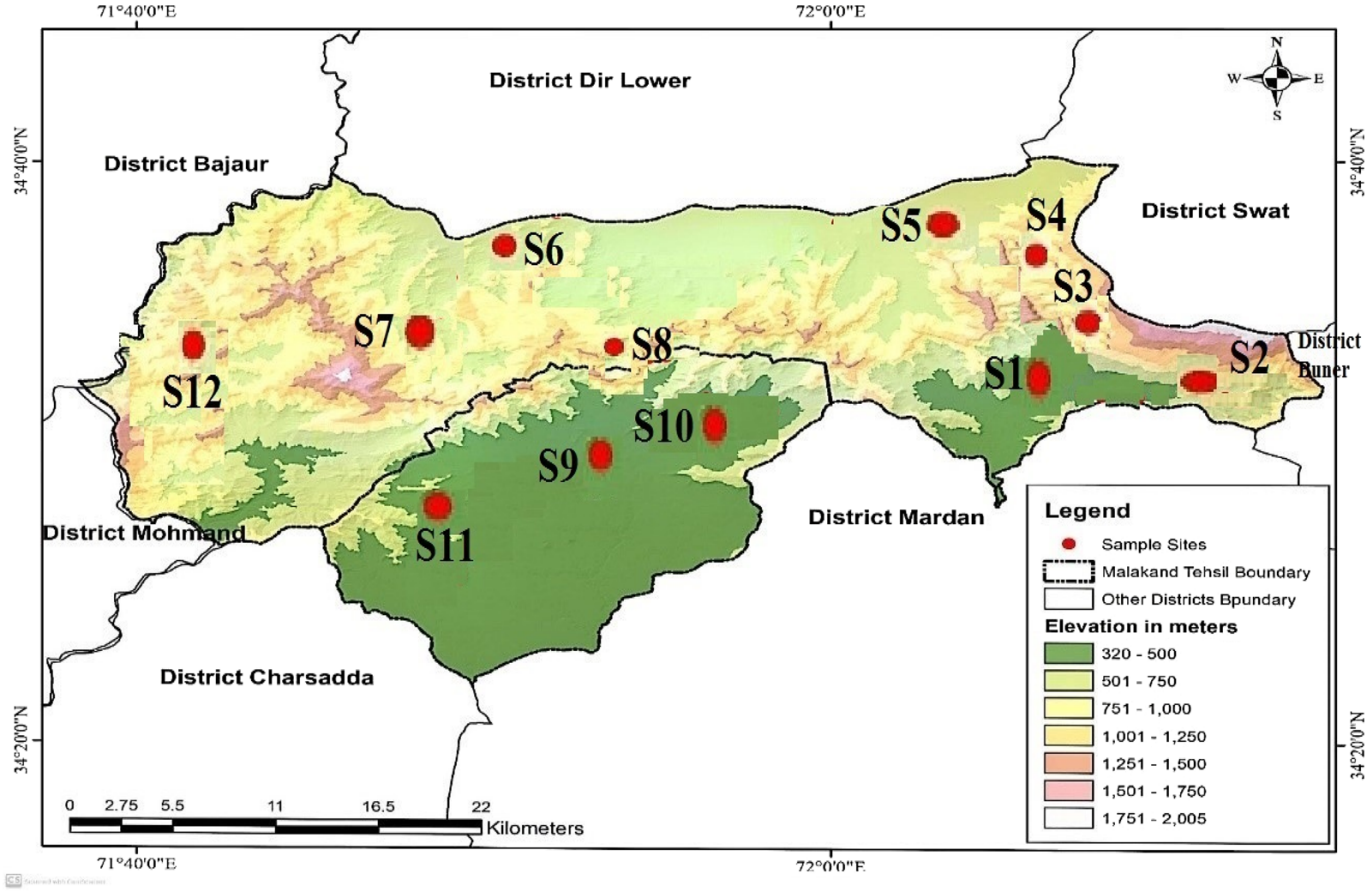
Map of the study area, generated by Arc GIS showing sampling sites (red dots)

District Malakand is the sub-tropical zone, situated to the northern side of Khyber Pakhtunkhwa, in the outer Hindukush mountains range (Ahmad et al. 2019). It is located 2705 feet above the sea level. District Malakand is geographically located 34° 35′ NL and 71° 57′ EL (Sciences 2022). It is enclosed by District Swat in the NE, District Dir (L) in the N, District Buner in the E, Districts Charsadda and Mardan in the S and Districts Bajaur and Mohmand in the W (Fig. 1).

### Vegetation sampling

For assessing vegetation, the area was divided into 12 representative stations viz; S1–S12 (Table 1). These stations were selected on the basis of altitude, aspect and landscapefeatures. The vegetation structure was analyzed using quadrat method such that quadrats of 1 m × 1 m were used for herbs, 5 m × 5 m for shrubs and 10 m × 10 m for trees.

**Table 1:** Topographic features of representative stations in District Malakand

Stations	Station name	Landscape	Altitude (m)	Altitude class	Aspect
S1	Palai	Plain	494–520	Low	–
S2	Bazdara	Plain	496–660	Low	–
S3	Palai-Thana	Hills	625–1050	High	South
S4	Palai-Thana	Hills	765–1072	High	North
S5	Thana	Plain	670–800	Middle	–
S6	Totakan	Plain	612–675	Middle	–
S7	Baika-Chapal	Hills	690–1110	High	North
S8	Malakand Pass	Hills	534–829	Middle	South
S9	Wartair	Plain	468–517	Low	–
S10	Jaban-Dargai	Watercourse	463–520	Low	–
S11	Palonao	Plain	430–454	Low	–
S12	Dheri-Kandao	Hills	501–688	Middle	South

### Physicochemical analyses of soil samples

About 1 kg soil samples up to 15 cm in depth were collected from each site. Physicochemical analyses of these soil samples were carried out in the Soil Science Laboratory of the Agricultural Research Institute (ARI), Tarnab, Peshawar. Soil texture was determined using a hydrometer method. The CaCO_3_ concentration was determined by acid neutralization method (Khan et al. 2017a, b; Jamil et al. 2022), organic matter was determined using the Walky-Black procedure (Ahmad et al. 2016; Rahman et al. 2016), soil nitrogen was determined using the Kjeldhal method (Rahman et al. 2016), soil pH was determined by testing a 1:5 soil: water suspension with a pH meter (Ali et al. 2017; Haq et al. 2022; Jamil et al. 2022), electrical conductivity was determined by testing a 1:5 soil: water suspension with a conductivity meter (Bano et al. 2018). Phosphorus and potassium were determined using the method described by Bano et al. (2018) and Khan et al. (2019b) respectively. Total soluble salts (TSS) were determined by the recommended method of (Evaluation et al. 2012). Soil moisture content was determined by the gravimetric method as Pohl et al. (2012) and De Vries et al. (2013).

### Data analysis

Various phytosociological procedures were used to assess the vegetation structure of the study area. Density, cover and frequency of each species was recorded and were converted into its relative values. The relative values of each parameter (Density, cover, frequency) for species were summed to get the importance values as (IV = R. D + R. C + R. F). Importance value of each species in a particular family was added together to give rise FIV for all the quantitatively recorded families. Biological spectra were determined using the approach of (Malik 2013; Majeed et al. 2022c, d). Similarity index was determined by using Sørensen similarity coefficient (Ali 2011). Shannon–Wiener diversity index (H $\mathrm{^{\prime}}$) was calculated following the method outlined in Shaheen et al. (2011b) and Jamil et al. (2022). The Simpson's diversity index was calculated according to the method given in Haq et al. (2022). Species richness was calculated using the formula provided in Gilchrist et al. (2018). The evenness index (J) and maturity index (MI) of sampled vegetation were calculated as per available literatures (Shaheen et al. 2011a, c). In order to determine the relationship of vegetation and environmental variables, canonical correspondence analysis (CCA) ordination for both species and sites were used (Leps and Smilauer 2006; Khan et al. 2020). Furthermore, the relationship of various environmental variables with diversity index (H'), species diversity index (SDI), species evenness (J), species richness (d), and maturity index (MI) of vegetation were emphasis by correlation and regression analysis via using SPSS (version 20). The homogeneity of the community (Bürzle et al. 2017) was calculated by using Raunkiaer’s law of frequency (Khan et al. 2013). The distribution pattern was calculated according to Ali et al. (2017).

## Results

### Vegetation diversity

**Fig. 2:**
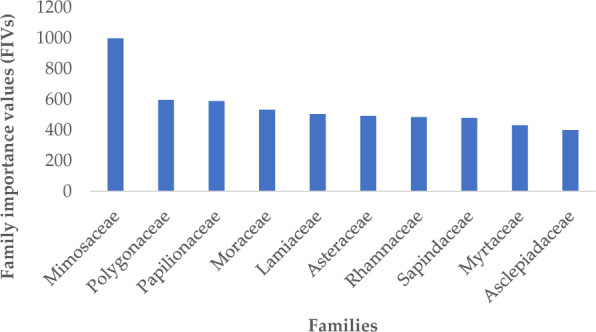
Leading plant communities based on family importance value FIV during spring

Quantitative analysis of vegetation during spring enlisted 252 species of 80 families in the study area. It included 39 species of trees, 43 species of shrubs, 167 species of herbs and 3 climber species. Based on FIVs, Mimosaceae (FIV = 998.07) was the leading family followed by Polygonaceae (FIV = 596.02), Papilionaceae (FIV = 588.44), Moraceae (FIV = 532.54), Lamiaceae (FIV = 504.15), Asteraceae (FIV = 492.33), Rhamnaceae (FIV = 484.65), Sapindaceae (FIV = 479.4), Myrtaceae (FIV = 431.58) and Asclepiadaceae (FIV = 399.72) (Fig. 2).

### Communities pattern

In the research area as a whole, twelve communities were established with its structural composition as:

#### Acacia-Lantana-Emex (ALE) community

ALE community was established in S1 at low altitude (494–520 m) having plain landscape. This community was formed by *Acacia nilotica*, *Lantana camara* and *Emexspinosus* with IV = 90.92, 76.35, 20.78 respectively (Table 2). This community comprised a total of 92 species (13 trees, 9 shrubs, 69 herbs and 1 climber). The associated species in tree stratum included *Eucalyptus camaldulensis*, *Ziziphus mauritiana* and *Acacia modesta*. *Calotropis procera*, *Ziziphus nummularia* and *Justicia adhatoda* were other important species in shrub stratum. *Arenaria serpyllifolia*, *Euphorbia helioscopia* and *Galium aparine* were other dominant species of herb stratum. The leading life form of ALE community was therophytes (60.9%) and the leaf size spectrum was dominated by nanophylls (42.4%) followed microphylls (33.7%) (Table 3). This community preferred to grow on sandy loam soil (54% sand, 44% silt, 2% clay). The soil was alkaline (8.1 pH) with 0.12 dS^−1^ EC. The other soil variables included 0.038% TSS, 7.5% CaCO_3_, 0.65% OM, 0.032 mg/kg N, 15.4 mg/kg P and 110 mg/kg K (Table 4).

**Table 2:** Quantitative aspects of vegetation from District Malakand

Communities*	T	S	H	Cl	TP	Dominant species	D	F	C	R. D	R. F	R. C	I. V
1. ALE	13	9	69	1	92	*Acacia nilotica*	2.1	100	18.30	30.43	25.64	34.84	90.92
						*Lantana camara*	2.8	100	20.50	28.00	20.41	27.94	76.35
						*Emexspinosus*	4.6	50	9.80	8.95	3.07	8.76	20.78
2. ADM	7	11	62	1	81	*Acacia modesta*	1.6	70	13.43	33.33	31.82	42.59	107.74
						*Dodonaeaviscosa*	2.1	90	17.95	22.11	17.65	26.53	66.29
						*Medicago minima*	5.7	70	8.70	10.75	4.96	8.02	23.74
3. AJM	5	16	41	1	63	*Acacia modesta*	1.7	80	16.11	48.57	40.00	58.43	147.00
						*Justicia adhatoda*	1.6	70	11.90	21.33	20.00	22.92	64.25
						Medicago minima	5.9	90	9.70	14.01	7.09	10.94	32.04
4. DPS	9	9	38	–	56	*Dodonaeaviscosa*	2.7	90	18.40	40.91	33.33	41.12	115.36
						*Pinus roxburghii*	1.5	60	18.77	36.59	28.57	49.79	114.95
						*Stellaria media*	8.9	90	19.00	19.47	8.57	21.81	49.85
5. CME	16	8	42	–	66	*Calotropis procera*	1.1	50	8.80	25.58	25.00	24.31	74.89
						*Melia azedarach*	1.5	80	11.58	19.74	14.55	23.25	57.54
						*Euphorbia helioscopia*	7.3	100	15.90	15.70	8.06	16.59	40.35
6. CMS	18	10	38	–	66	*Calotropis procera*	1.0	50	10.80	18.87	21.74	24.40	65.01
						*Melia azedarach*	2.2	100	16.14	20.18	14.93	24.51	59.62
						*Stellaria media*	6.8	100	12.30	14.32	8.55	13.12	35.99
7. PDT	17	19	39	1	76	*Pinus roxburghii*	1.8	90	9.22	19.15	24.66	19.64	63.44
						*Dodonaeaviscosa*	2.3	80	17.40	23.00	14.55	24.98	62.53
						*Tulipa clusiana*	6.2	60	10.30	13.11	6.45	11.40	30.96
8. ADC	14	15	23	1	53	*Acacia modesta*	1.5	70	9.73	27.27	17.50	29.90	74.67
						*Dodonaeaviscosa*	1.9	70	11.80	24.36	14.89	20.52	59.78
						*Cynodondactylon*	3.8	90	12.90	13.67	12.68	17.92	44.26
9. SPS	15	14	34	–	63	*Saccharum bengalense*	0.8	40	7.20	13.33	16.67	16.76	46.76
						*Populus nigra*	1.5	80	9.16	14.71	11.94	12.17	38.82
						*Silybum marianum*	7.9	90	13.90	15.46	6.72	14.07	36.24
10. VLA	12	11	36	–	59	*Vitex negundo*	2.4	90	189.50	27.27	30.38	31.61	89.26
						*Leucaena leucocephala*	2.5	100	12.00	23.58	19.23	18.34	61.16
						*Agrostis viridis*	6.7	100	23.50	15.95	7.94	24.96	48.85
11. ZME	13	9	46	–	68	*Ziziphus nummularia*	1.6	60	12.30	24.24	28.57	28.94	81.76
						*Morus nigra*	1.2	90	10.94	13.48	15.00	17.26	45.74
						*Emexspinosus*	4.5	90	10.80	11.00	7.32	11.92	30.24
12. ADA	14	12	60	–	86	*Acacia modesta*	1.8	70	10.85	30.00	19.44	37.00	86.44
						*Dodonaeaviscosa*	3.0	100	19.50	29.13	20.83	30.65	80.61
						*Arenaria serpyllifolia*	4.3	70	5.60	9.27	4.83	5.87	19.97

Key to communities*: ALE = *Acacia-Lantana-Emex*, ADM = *Acacia-Dodonaea-Medicago*, AJM = *Acacia-Justicia-Medicago*, DPS = *Dodonaea-Pinus-Stellaria*, CME = *Calotropis-Melia-Euphorbia*, CMS = *Calotropis-Melia-Stellaria*, PDT = *Pinus-Dodonaea-Tulipa*, ADC = *Acacia-Dodonaea-Cynodon*, SPS = *Saccharum-Populus-Silybum*, VLA = *Vitex-Leucaena-Agrostis*, ZME = *Ziziphus-Morus-Emex*, ADA = *Acacia-Dodonaea-Arenaria*

**Table 3:** Biological spectrum of plant communities in the studied area, District Malakand

Communities*	ALE	ADM	AJM	DPS	CME	CMS	PDT	ADC	SPS	VLA	ZME	ADA
*A. Life form spectrum*												
Therophytes	60.9	56.8	30.2	35.7	54.5	47	27.6	30.2	49.2	42.4	58.8	45.3
Chamaephytes	1.1	3.7	6.3	1.8	1.5	1.5	3.9	7.5	1.6	3.4	1.5	9.3
Hemicryptophytes	8.7	12.3	27	26.8	4.5	7.6	15.8	7.5	3.2	13.6	8.8	14
Geophytes	4.3	4.9	1.6	3.6	3	1.5	5.3	0	1.6	1.7	0	2.3
Nanophanerophytes	8.7	12.3	22.2	10.7	7.6	12.1	19.7	22.6	14.3	11.9	10.3	8.1
Microphanerophytes	2.2	1.2	4.8	5.4	4.5	3	5.3	7.5	6.3	6.8	1.5	4.7
Mesophanerophytes	4.3	2.5	3.2	7.1	10.6	10.6	11.8	11.3	9.5	6.8	4.4	7.0
Megaphanerophytes	9.8	6.2	4.8	8.9	13.6	16.7	10.5	13.2	14.3	13.6	14.7	9.3
*B. Leaf size spectrum*												
Leptophylls	7.6	11.1	12.7	12.5	3	4.5	6.6	5.7	6.3	3.4	7.4	7.0
Nanophylls	42.4	40.7	30.2	33.9	43.9	34.8	31.6	28.3	34.9	25.4	33.8	34.9
Microphylls	33.7	35.8	42.9	39.3	28.8	33.3	43.4	39.6	27	42.4	35.3	41.9
Mesophylls	15.2	12.3	11.1	14.3	22.7	24.2	15.8	24.5	31.7	27.1	22.1	15.1
Megaphylls	0	0	0	0	0	1.5	1.3	0	0	0	0	1.2
Aphyllous	1.1	0	3.2	0	1.5	1.5	1.3	1.9	0	1.7	1.5	0

**Table 4:** Environmental variables of the studied area, District Malakand

Station	Clay	Silt	Sand	Texture class	pH	MC %	EC dsm^−1^	TSS	CaCO_3_	OM	N	P	K
	-----%-----							-----%-----			-----mg/kg-----		
1. Palai	2	44	54	Sandy loam	8.1	5.3	0.12	0.038	7.5	0.65	0.032	15.4	110
2. Bazdara	2	40	58	Sandy loam	8.1	4.2	0.11	0.035	6.8	0.72	0.036	8.0	120
3. Palai Thana	4	36	60	Sandy loam	8.0	4.5	0.11	0.035	10	0.72	0.036	5.4	95
4. Palai Thana	2	38	60	Sandy loam	8.0	6.8	0.11	0.035	8.8	0.69	0.034	2.2	110
5. Thana	4	42	54	Sandy loam	8.0	9.1	0.12	0.038	7.5	0.79	0.039	7.7	110
6. Totakan	6	36	58	Sandy loam	8.0	4.5	0.11	0.035	8.3	0.69	0.034	14.8	120
7. Baika Chapal	8	44	48	Loam	8.0	12.5	0.11	0.035	6.8	0.86	0.043	22.8	98
8. Malakand Pass	2	34	64	Sandy loam	8.0	5.7	0.12	0.038	8.0	0.82	0.041	19.4	100
9. Wartair	10	42	48	Loam	7.9	6.7	0.10	0.032	9.0	0.86	0.043	6.2	140
10. Jaban-Dargai	2	38	60	Sandy loam	8.1	20.9	0.20	0.064	7.5	0.69	0.034	4.0	110
11. Palonao	4	44	52	Sandy loam	8.5	6.5	0.20	0.064	7.8	0.79	0.039	5.7	90
12. Dheri-Kandao	6	32	62	Sandy loam	8.1	5.9	0.11	0.035	8.5	0.65	0.032	4.2	96

#### Acacia-Dodonaea-Medicago (ADM) community

ADM community was established at S2 at 496–660 m altitude in plain landscape. This community was dominated by *Acacia modesta* (IV = 107.74) in tree stratum, *Dodonaea viscosa* (IV = 66.29) in shrub stratum and *Medicago minima* (IV = 23.74) in ground stratum (Table 2). This community included a total of 81 species (7 trees, 11 shrubs, 62 herbs and 1 climber). *Eucalyptus camaldulensis* and *Ziziphus* mauritiana were codominant species of tree stratum. *Saccharum griffithii*, *Justicia adhatoda* and *Sageretia thea* were the important species in shrub stratum. *Bromus pectinatus*, *Calendula arvensis* and *Anagallis arvensis* were the other important herbs. The life form spectrum of ADM community was dominated by therophytes (56.8%). The leaf size spectrum was dominated by nanophylls (40.7%) followed by microphylls (35.8%) (Table 3). Soil textural class was sandy loam with 58% sand, 40% silt and 2% clay. Moisture contents was 4.2%. Soil pH value was 8.1, EC 0.11 dSm^−1^, TSS 0.035%. The least CaCO_3_ was found for this community which was 6.8%. Soil organic matter was 0.72%, N 0.036 mg/kg, P 8.0 mg/kg and K 120 mg/kg (Table 4).

#### Acacia-Justicia-Medicago (AJM) community

AJM community was established at S3 in the south aspect of the hilly landscape at high altitude (625–1050 m). This community was comprised a total of 63 species (5 trees, 16 shrubs, 41 herbs and 1 climber). This community was dominated by *Acacia modesta* in tree stratum, *Justicia adhatoda* in shrub stratum and *Medicago minima* in herbaceous stratum having IV of 147.00, 64.25, 32.04 respectively (Table 2). The codominant tree species were *Monotheca buxifolia* and *Acacia nilotica* while *Dodonaea viscosa*, *Otostegia limbata* and *Sageretia thea* were the codominant shrubs. The codominant species in herb stratum were *Arenaria serpyllifolia*, *Erodium* malacoides and *Lactuca dissecta*. The life form spectrum of AJM community was dominated by therophytes (30.2%), followed by hemicryptophytes (27.0%). The leaf size spectrum showed microphylls (42.9%) and nanophylls (30.2%) as the leading leaf size classes (Table 3). This community preferred to grow on sandy loam soil with 60% sand, 36% silt and 4% clay. Moisture contents was 4.5%, pH 8.0, EC 0.11 dsm^−1^ and TSS 0.035%, OM 0.72%, N 0.036 mg^−kg^, P 5.4 mg^−kg^ and K 95 mg ^−kg^. The maximum CaCO_3_ (10.0%) was observed in soil samples of this community (Table 4).

#### Dodonaea-Pinus-Stellaria (DPS) community

DPS community was established in S4 at north aspect of hilly landscape at high altitude (756–1072 m). This community was composed of a total of 56 species (9 trees, 9 shrubs and 38 herbs). The dominant species were *Dodonaeaviscosa* in shrub stratum, *Pinus roxburghii* in tree stratum and *Stellaria media* in herb stratum with IVs of 115.36, 114.95, 49.85 respectively (Table 2). *Eucalyptus camaldulensis* and *Ailanthus altissima* in tree stratum while *Otostegia limbata*, *Mallotus philippensis* and *Daphne mucronata* in shrub stratum were the dominant species. *Arabidopsis thaliana*, *Micromeria biflora* and *Nepeta griffithii* were the dominant species of the herb stratum. The life form spectrum of DPS community was dominated by therophytes (35.7%) followed by hemicryptophytes (26%). The leaf size spectrum was dominated by microphylls (39.3%) followed by nanophylls (33.9%) (Table 3). The soil texture of this community was sandy loam with 60% sand, 38% silt and 2% clay. Moisture contents was 6.8%, pH 8.0, EC 0.11 dSm^−1^, TSS 0.035%, CaCO_3_ 8.75%, OM 0.69%, N 0.034 mg ^−kg^, K 110 mg ^– kg^. The least P was found for this community which was 2.2 mg ^−kg^ (Table 4).

#### Calotropis-Melia-Euphorbia (CME) community

CME community was established at S5 in plain landscape at middle altitude (670–800 m). This community was dominated by *Calotropis procera*, *Melia azedarach* and *Euphorbia helioscopia* having IVs of 74.89, 57.54, 40.35 respectively (Table 2). This community included a total of 66 species (16 trees, 8 shrubs and 42 herbs). The codominant species among trees included *Robinia pseudo-acacia* (IV = 49.56), *Ailanthus altissima* (IV = 37.23) and *Populus nigra* (IV = 34.36). The codominant species among shrubs included *Rosa multiflora*, *Vitex negundo* and *Ziziphus nummularia*. The codominant species among herbs included *Poa annua*, *Taraxicum officinale* and *Morea sisyrinchium*. The life form spectrum of CME community was dominated by therophytes (54.5%). The leaf size form spectrum was dominated by nanophylls (43.9%) followed by microphylls (28.8%) (Table 3). The soil samples of this community contained 54% sand, 42% silt and 4% clay. Moisture contents was relatively high (9.1%). Soil pH was 8.0, EC 0.12 dSm^−1^, TSS 0.038%, CaCO_3_ 7.5%, OM 0.79%, N 0.039 mg ^−kg^, P 7.7 mg ^– kg^ and K 110 mg ^– kg^ (Table 4).

#### Calotropis-Melia-Stellaria (CMS) community

CMS community was developed at S6 in plain landscape at middle altitude (612–675 m). This community was dominated by *Calotropis procera*, *Melia azedarach* and *Stellaria media* having IVs of 65.01, 59.62, 35.99 respectively (Table 2). This community included a total of 66 species (18 trees, 10 shrubs and 38 herbs). The associated trees species included *Populus nigra*, *Ailanthus altissima* and *Morus nigra* among trees while the associated shrubs species included *Vitex negundo*, *Ziziphus nummularia* and *Lantana camara*. *Emexspinosus*, *Centaurea iberica* and *Euphorbia helioscopia* were the associated herbs. The life form spectrum of CMS community was dominated by therophytes (47.0%). The leaf size spectrum was dominated by nanophylls (34.8%) followed by microphylls (33.3%) (Table 3). Soil texture was sandy loam with sand (58%), silt (36%) and clay (6%). Moisture contents was less (4.5%), pH 8.0. EC was 0.11 dSm^−1^, TSS 0.035%, CaCO_3_ 8.3%, OM 0.69%, N 0.034 mg ^−kg^. The soil samples contained high quantity of P (14.8 mg ^– kg^) and K 120 mg ^−kg^ (Table 4).

#### Pinus-Dodonaea-Tulipa (PDT) community

PDT community was established at S7 in the north aspect of the hilly landscape at high altitude (690–1110 m). This community was dominated by *Pinus roxburghii*, *Dodonaea viscosa* and *Tulipa clusiana* having IVs of 63.44, 62.53, 30.96 respectively (Table 2). This community included a total of 76 species (17 trees, 19 shrubs, 39 herbs and 1 climber). The associated species in tree layer included *Olea ferruginea*, *Pyrus pseudopashia* and *Phoenix sylvestris*. The associated shrubs included *Rubus fruticosus*, *Saccharum griffithii* and *Myrsine africana* while the associated herbs included *Plantago lanceolata*, *Scilla griffithii* and *Bromus pectinatus*. The life form spectrum of PDT community was dominated by therophytes (27.6%) followed by nanophanerophytes (19.7%). The leaf size spectrum was dominated by microphylls (43.4%) followed by nanophylls (31.6%) (Table 3). Soil texture of this community was loam with 48% sand, 44% silt and 8% clay. Moisture contents was higher (12.5%). Soil pH was 8.0, EC 0.11 dSm^−1^, TSS 0.035%. The least CaCO_3_ (6.8%) while maximum OM (0.86%), N (0.043 mg ^– kg^) and P (22.8 mg ^– kg^) was observed in soil samples of this community. The soil samples contained 98 mg ^−kg^ Potassium (Table 4).

#### Acacia-Dodonaea-Cynodon (ADC) community

ADC community was established at S8 in south aspect of hilly landscape at middle altitude (534–829 m). This community was formed by *Acacia modesta*, *Dodonaea viscosa* and *Cynodon dactylon* having IVs of 74.67, 59.78, 44.26 respectively (Table 2). This community included a total of 53 species including 14 trees, 15 shrubs, 23 herbs and 1 climber. The associated species included *Olea ferruginea*, *Eucalyptus camaldulensis* and *Dalbergia sissoo* among trees. The associated species in shrub stratum included *Justicia adhatoda*, *Cocculus pendulus* and *Saccharum griffithii*. The associated herbs species included *Lactuca dissecta* and *Farsetia jacquemontii* in herbs. The life form spectrum of ADC community was dominated by therophytes (34.0%) and hemicryptophytes (21.6%). The leaf size spectrum was dominated by microphylls (39.6%) followed by nanophylls (28.3%) (Table 3). Soil of this community was sandy loam with 64% sand,34% silt and 2% clay. Moisture contents was 5.7%, pH 8.0, EC 0.12 dSm^−1^, TSS 0.038%, CaCO_3_ 8.0%, OM 0.82%, N 0.041 mg/kg, P 19.4 mg/kg and K 100 mg/kg (Table 4).

#### Saccharum-Populus-Silybum (SPS) community

SPS community was established at S9 in plain landscape at low altitude (468–517 m). This community was dominated by *Saccharum bengalense*, *Populus nigra* and *Silybum marianum* with IVs of 46.76, 38.82, and 36.24 respectively (Table 2). This community included a total of 63 species (15 trees, 14 shrubs and 34 herbs). *Morus nigra*, *Melia azedarach* and *Broussonetia papyrifera* among trees were the dominant species. *Calotropis procera*, *Ipomoea carnea* and *Rosa multiflora* among shrubs were the dominant species while *Euphorbia helioscopia*, *Stellaria media* and *Phalaris minor* were other important species among herbs. The life form spectrum of SPS community was dominated by therophytes (42.7%). Nanophylls (34.9%) and mesophylls (31.7%) dominated the leaf size spectrum of SPS community (Table 3). Soil of this community was loam with 48% sand, 42% silt and 10% clay. Minimum pH value was recorded in the soil samples of this community (pH = 7.9). Moisture contents was 6.7%, EC 0.10 dSm^−1^, TSS 0.032%. The maximum CaCO_3_, (9.0%), OM (0.86%) N (0.043 mg/kg) and K (140 mg/kg) was recorded in the soil samples of this community while P was 6.2 mg/kg (Table 4).

#### Vitex-Leucaena-Agrostis (VLA) community

VLA community was established at S10 in watercourse landscape at low altitude (463–520 m). This community was dominated by *Vitex negundo*, *Leucaena leucocephala* and *Agrostis viridis* having IVs of 89.26, 61.16, 48.85 respectively (Table 2). This community included a total of 59 species (12 trees, 11 shrubs and 36 herbs). *Populus nigra*, *Eucalyptus camaldulensis* and *Broussonetia papyrifera* among trees were the other dominant species while *Arundo donax*, *Ipomoea carnea* and *Debregeasia salicifolia* among shrub stratum. *Mentha longifolia* and *Nasturtium officinale* were other dominant species of herb stratum. The life form spectrum of VLA community was dominated by hemicryptophytes (23.0%) followed by therophytes (21.3%). The leaf size spectrum was dominated by microphylls (42.4%) followed by mesophylls (27.1%) (Table 3). The soil texture of this community contained sand (60%), silt (38%) and clay (2%). The maximum moisture contents (20.9%) was observed in soil samples of this community. This community showed soil pH value of 8.1, EC 0.20 dSm^−1^, TSS 0.064%, CaCO_3_ 7.5%, OM 0.69 mg/kg, N 0.034 mg/kg, P 4.0 mg/kg and K 110 mg/kg (Table 4).

#### Ziziphus-Morus-Emex (ZME) community

ZME community was established at S11 in plain landscape at low altitude (430–454 m). This community was dominated by *Ziziphus nummularia*, *Morus nigra* and *Emex spinosus* with IVs of 81.76, 45.74, 30.24 respectively. This community included a total of 68 species (13 trees, 9 shrubs and 46 herbs) (Table 2). The other associated species among trees included *Acacia nilotica*, *Populus nigra* and *Melia azedarach*. The other dominant species among shrubs included *Calotropis procera*, *Ipomoea carnea* and *Saccharum bengalense* while herbs included *Euphorbia helioscopia*, *Medicago polymorpha* and *Salvia moorcroftiana*. The life form spectrum of ZME community was dominated by therophytes (49.3%). The leaf size spectrum was dominated by microphylls (35.3%) followed by nanophylls (33.8%) (Table 3). The soil samples of this community had 54% sand, 44% silt and 4% clay with maximum pH (8.5). Moisture contents was 6.5%. EC 0.20 dSm^−1^, TSS 0.064%, CaCO_3_ 7.8%, OM 0.79%, N 0.039 mg/kg, P 5.7 mg/kg and K 90 mg/kg (Table 4).

#### Acacia-Dodonaea-Arenaria (ADA) community

ADA community was established at S12 in south aspect of hilly landscape at middle altitude (501–688 m). This community was dominated by *Acacia modesta*, *Dodonaea viscosa* and *Arenaria serpyllifolia* with IVs of 86.44, 80.61, 19.97 respectively. This community included a total of 86 species (14 trees, 12 shrubs and 60 herbs) (Table 2). *Olea ferruginea*, *Ailanthus altissima* and *Eucalyptus camaldulensis* were associated species in tree stratum. *Justicia adhatoda*, *Otostegia limbata* and *Colebrookea oppositifolia* in shrub stratum while *Micromeriabiflora* and *Ajuga bracteosa* were associated species in herb stratum. The life form spectrum of ADA community was dominated by therophytes (45.3%). The leaf form spectrum was dominated by microphylls (41.9%) followed by nanophylls (34.9%) (Table 3). The soil texture contained 62% sand, 32% silt and 6% clay. Moisture contents was 5.9%, pH 8.1, EC 0.11dSm^−1^, TSS 0.035%, CaCO_3_ 8.50%, OM 0.65%, N 0.032 mg/kg, P 4.2 mg/kg and K 96 mg/kg (Table 4).

### Diversity indices

In the present study, the Shannon–Weaver diversity index (H′) and Simpson diversity index varied from 3.30 to 3.98 and 0.02 to 0.06 respectively (Table 5). The highest H′ (3.98) was recorded at S1. It was followed by S12 (H′, 3.94). Similarly, H′ values recorded in S2 and S11 were respectively 3.86 and 3.83. The lowest species diversity index values (H′ = 3.30) were found for S4, S8 (H′ **= **3.50) and S10 (H′ = 3.53). These results are also verified by Simpson diversity index. The values of the evenness index varied from 0.82 to 0.91 (Table 5). The highest value of evenness index (0.91) was found in S11. It was followed by S1, S2, S8, S12, these all stations had evenness value of 0.88. The lowest evenness index was found in S4 and S3 that was respectively 0.82 and 0.86. The values of species richness index ranged from 9.00 to 14.10 (Table 5). The maximum species richness index (14.10) was found in S1. It was followed by S12 and S2 with 13.35 and 12.44 respectively. The lowest species richness index (8.81) was recorded in S8 and 9.00 in S4. The values of maturity index ranged from 25.66 to 35.76 (Table 5). The maximum maturity index (35.76) was found in S10 followed by S9 (35.71) and S6 (31.36). The least MI value (25.66) was found in S8 followed S2 (26.42) and S12 (26.63). The similarity was highest (39.8%) between S1 and S11. The second highest similarity (38.3%) was recorded between S5 and S6. On the other hand, the least similarity (10.2%) was recorded between S3 and S10 (Table 6).

**Table 5:** Diversity index, evenness, species richness and maturity index of the spring vegetation

Indices/stations	S1	S2	S3	S4	S5	S6	S7	S8	S9	S10	S11	S12
Diversity index (H')	3.98	3.86	3.60	3.30	3.64	3.65	3.78	3.50	3.62	3.53	3.83	3.94
Diversity index (SDI)	0.98	0.97	0.96	0.94	0.96	0.96	0.97	0.96	0.96	0.96	0.97	0.97
Evenness (J)	0.88	0.88	0.86	0.82	0.87	0.87	0.87	0.88	0.87	0.87	0.91	0.88
Species richness (d)	14.10	12.44	10.19	9.00	10.36	10.22	11.75	8.81	9.67	9.21	10.73	13.35
Maturity index (MI)	27.28	26.42	28.44	26.84	30.15	31.36	25.66	29.81	35.71	35.76	30.00	26.63

**Table 6:** Similarity among different stations of the studied area

Stations	S1	S2	S3	S4	S5	S6	S7	S8	S9	S10	S11	S12
S1	X											
S2	36.6	X										
S3	27.9	28.7	X									
S4	22.0	28.9	27.7	X								
S5	31.3	32.3	14.6	23.6	X							
S6	30.1	32.9	20.9	31.3	38.3	X						
S7	21.5	30.2	22.4	33.8	22.0	27.6	X					
S8	30.0	26.4	30.1	26.7	26.1	26.1	18.9	X				
S9	35.7	32.7	18.2	17.8	34.5	31.0	20.6	27.5	X			
S10	21.8	22.2	10.3	18.3	27.8	22.4	19.2	23.3	33.0	X		
S11	39.1	36.8	23.5	23.5	36.4	34.5	24.3	26.8	25.9	25.0	X	
S12	29.9	34.5	32.0	32.9	24.0	30.9	29.6	30.6	23.6	22.5	33.8	X

### CCA ordination and influence of environmental variables on diversity index (H'), species diversity index (SDI), evenness (J), species richness (d) and maturity index (MI)

**Fig. 3:**
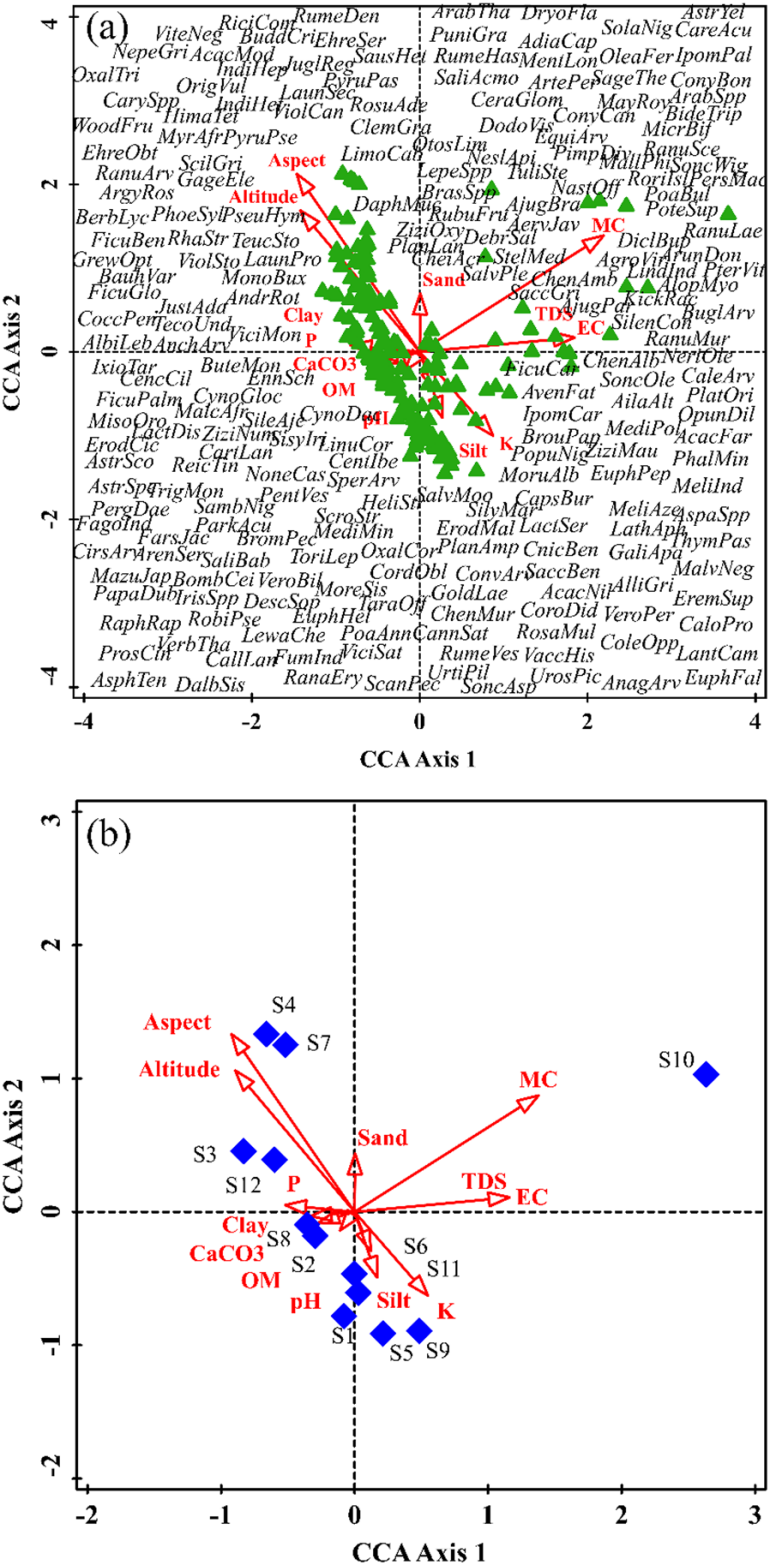
Canonical Correspondence Analysis (CCA) ordination biplot of **a** 252 species, and **b** sites ordination based on influence of various environmental variables

The environmental variables, namely pH, EC, TDS, CaCO3, OM, P, K, clay, silt, sand, MC, altitude and aspect, play a significant role in shaping vegetation and its characteristics, such as diversity index, species diversity index, evenness, richness, and maturity index. The red arrow in the CCA ordination biplot represent the environmental variables, while the green triangle demonstrates the species (Fig. 3a). Similarly, the blue diamond represents the sampled sites (Fig. 3b). The length of arrow in the ordination biplot demonstrates the dimension between various environmental variables and their correlation. Variables aligned along the same axis indicate a positive relationship, whereas those positioned in opposite directions reveals a negative relationship. The CCA ordination demonstrates the species environment-relationship for axes 1 (0.988), 2 (0.963), 3 (0.937), and 4 (0.911) (Table 7). The sum of all eigenvalues and canonical eigenvalues were 10.833 and 2.776 respectively. The permutation test demonstrated *F* (3.756) and *p* (0.002). The CCA ordination biplot demonstrates that vegetation in the first quadrant is primarily influenced by sand, MC, TDS and EC. The vegetation of second quadrant of CCA is associated with aspect, altitude, and phosphorus (P). The vegetation in the third quadrant shows association with clay, CaCO_3,_ OM and pH, whereas silt and potassium (K) are associated with vegetation in the fourth quadrant of CCA ordination.

**Table 7:** Summary of CCA ordination of vegetation and environmental variables of the study area

Axes	1	2	3	4
Eigenvalues	0.640	0.490	0.424	0.277
Species environment relationship	0.988	0.963	0.937	0.911
Cumulative percentage variance of species	5.901	10.401	14.410	16.921
Cumulative percentage variance of species-environment relationship	23.120	40.701	56.011	66.001
Sum of all eigenvalues:				10.833
Sum of all canonical eigenvalues:				2.776
			Test of significance of all canonical axes	
Eigenvalue	0.988		Trace	2.776
*F* ratio	1.423		*F*-ratio	3.756
*p* value	0.005		*P*-value	0.002

The influence of environmental variables on vegetation was further elaborated through correlation and linear regression analysis. EC, TDS, K, and MC show a significant positive correlation with vegetation, whereas phosphorus, altitude and aspect reveal a significant negative relationship with vegetation (Table 8). pH and phosphorus showed a significant positive relationship with the diversity index and species evenness. However, species richness and maturity index did not show a relationship with pH. EC, while TDS revealed a significant positive relationship with species evenness and maturity index. Potassium and clay show a positive correlation with species diversity index and maturity index (Table 8). Sand and altitude showed a significant positive correlation with species diversity index and maturity index. CaCO3 demonstrates a significant negative relationship with the diversity index, species evenness, species evenness and species richness. Similarly, aspect showed a significant negative association with the maturity index.

**Table 8:** Correlation and regression analysis of environmental variables with vegetation, diversity index (H'), species diversity index (SDI), species evenness (J), species richness (d) and species maturity index (MI)

Environmental variables	Average	SDE	VIFE	Correlation (*r*)	Correlation (*r*) with Diversity index (H')	Correlation (*r*) with species Diversity index (SDI)	Correlation (*r*) with Species Evenness (J)	Correlation (*r*) with Species richness (d)	Correlation (*r*) with Maturity index (MI)
pH	8.08	0.15	413.92	0.071	0.414**	0.390*	0.646^**^	0.229	− 0.101
EC	0.12	0.04	286.72	0.641**	− 0.014	0.079	0.430**	− 0.229	0.463**
TDS	0.027	0.04	0.34	0.641**	− 0.014	0.079	0.430**	− 0.229	0.463**
CaCO3	7.98	0.88	50.28	− 0.182	-.405**	− 0.480**	− 0.385*	− 0.361*	0.198
OM	0.76	0.08	10.90	− 0.062	− 0.018	0.078	0.216	− 0.199	0.208
P	9.73	6.34	11.16	− 0.293*	0.313*	0.344*	0.294*	0.473**	0.113
K	108.28	13.54	55.12	0.312*	0.213	0.392*	0.226	0.176	− 0.261
Clay	4.41	2.57	12.71	− 0.151	− 0.165	− 0.192	− 0.242	− 0.149	0.505**
Silt	39.51	4.01	14.64	0.103	0.160	0.092	0.089	0.035	0.226
Sand	56.09	4.98	0.57	0.012	0.284	0.343*	0.234	0.214	0.014
MC	7.64	4.46	157.98	0.764***	− 0.304*	− 0.316	− 0.229	− 0.186	− 0.126
Altitude	637.20	167.17	15.31	− 0.495**	− 0.235	− 0.093	− 0.064	− 0.273	0.443**
Aspect	1.53	0.74	13.18	− 0.513**	− 0.450**	− 0.494**	− 0.700^*^	− 0.212	− 0.529**

^*^ = 0.05, ** = 0.01, *** = 0.001

## Discussion

The composition and diversity pattern of plant species are usually attributed to edaphic and environmental factors (Khan et al. 2017a, b; Rahman et al. 2021). In the present study a total of 252 species of 80 families were enlisted in the study area. It included 39 species of trees, 43 shrubs, 167 species of herbs and 3 climber species. Based on FIVs, the important families included Mimosaceae, Polygonaceae, Papilionaceae, Moraceae, Lamiaceae, Asteraceae, Rhamnaceae, Sapindaceae, Myrtaceae and Asclepiadaceae. Mimosaceae is virtually always the most diverse family throughout the subcontinent (Sher et al. 2011; Wariss et al. 2014). Papilionaceae has a dominant position in plant families (Zeb et al. 2017). Asteraceae, with more than 1620 genera and 23,600 species of herbs, shrubs and trees distributed throughout the world and is also one of the largest plant family (Carvalho et al. 2018). Similarly, Moraceae and Mimosaceae were also the important families in the present findings which agreed with the studies of Murad et al. (2013) and Ullah et al. (2016). The ranges of altitude have an important effect on qualitative and quantitative characteristics of species (Balick 2005; Arshad et al. 2014). As a whole, 12 communities were established on the basis of topographic and edaphic characteristics in 12 different stations.

In the current study, the dominant shrubs reported in various communities were *Calotropis procera*, *Dodonaeaviscosa*, *Justicia adhatoda, Lantana camara, Saccharum bengalense, Vitex negundo* and *Ziziphus nummularia*. In the current study, *Dodonaeaviscosa* was reported from 5 communities and *Calotropis procera* from 2 communities. The present findings are in line with (Haq et al. xxxx; Arshad et al. 2022) who reported *Dodonaea viscosa* as dominant species. Who (Majeed et al. 2021a) reported *Calotropis procera* as the dominant shrub species. Who (Qureshi et al. 2009; Majeed et al. 2021a) also found *Saccharum* as one of the dominant species. They (Ilyas et al. 2013, 2018) reported *Vitex negundo*as and *Ziziphus* dominant members of various plants communities in their research areas.

In the investigated attempt showed the dominant shrubs reported in various communities were *Agrostis viridis, Arenaria serpyllifolia*,* Cynodondactylon, Emexspinosus, Euphorbia helioscopia, Medicago minima,Silybum marianum*,* Stellaria media* and *Tulipa clusiana*. *Emexspinosus Medicago minima* and *Stellaria media* were recorded from 2 communities. The present findings agreed with (Sharma et al. 2017; Baruah et al. 2019) who recorded *Stellaria media* as a most commonly occurring species. Iqbal et al. (2017) reported *Emexspinosus*as the dominant species. They (Ahmad et al. 2017; Shinwari et al. 2017) reported *Medicago minima* as the dominant species of the vegetation around Havalian. Who (Zereen and Sardar 2015) recorded *Euphorbia helioscopia* as dominant species in a sub-community in Narowal district Punjab. They (Haq et al. xxxx; Zahoor et al. 2015) also reported *Cynodon dactylon* as the leading member of the plant community. Similarly, Ali et al. (2018) reported *Arenaria serpyllifolia* as the first dominant species in various communities from their studied area.

Plant life-form is a measurable trait and is also regarded as a potential indicator of prevailing environmental conditions (Gravel et al. 2006; Aubin et al. 2008). The leading life forms of the recorded communities were therophytes and hemicryptophytes. The therophytes as the dominant life form in the study area indicate human disturbances. The present findings also reported the dominance of therophytes like other studies (Hussain et al. 2015; Khan et al. 2018) in their studies. Severe deforestation, overgrazing, soil erosion and human influence in the area reduces the phanerophytes and so therophytes appear to occupy the vacant niches (Dar et al. 2018; Haq et al. 2019; Abdo 2021). Evaluating leaf size spectra, an important ecological trait can help to understand climatic factors that structure the plant communities (Majeed et al. 2021c, 2022b). The leaf size spectrum of the present plant communities showed that the overall vegetation of the study area is dominated by microphylls and nanophylls. The results are in agreement with Majeed et al. (2020, 2022c, d) and Ullah et al. (2022a, b).

Species diversity is a significant character of vegetation that reflects the productivity and health of ecosystem which is under the control of complex of environmental variables (Khan et al. 2022; Tassadduq et al. 2022). The range of diversity indices values of the present communities are in line with previous literatures (Khoja et al. 2022; Majeed et al. 2022a), there was very less similarity in plant communities between the low elevation station and high elevation stations.

Environmental variables play a significant role in shaping species distribution and abundance (Khan et al. 2017a, b; Ullah et al. 2022a, b; Majeed et al. 2022b). To determine the influence of environmental variables on the vegetation in our study, we employed CA ordination, correlation and linear regression analysis. The CCA ordination of species and sites demonstrates that EC, TDS, MC, altitude, and aspect have significantly influence the vegetation in our study area. Similarly, a study in the Yakhtangay Hill of Shangala district, Pakistan yielded comparable results (Ullah et al. 2022a, b). This implies that the distribution pattern of species is primarily influenced by the environmental variables of an area (Khan et al. 2020; Iqbal et al. 2022; Rawat et al. 2022). The correlation and linear regression analysis revealed that pH, phosphorus, potassium and sand shows positive association with diversity indices, whereas pH, EC, TDS and phosphorus are strongly linked to species evenness. Among the environmental variables, EC, TDS, clay and altitude showed a positive association with the maturity index. Phosphorus is the only key environmental variable that exhibits a positive association with both species richness and evenness. The possible reason may be the high content of phosphorus in the study area. A high content promotes growth and development, creating more available niches and resources for other species. This increase in ecological niches and resources leads to increase in species richness and ultimately spices evenness. Similar to our study, other study also shown that environmental variables, particularly edaphic factors (soil properties) and topographic factors (such as slope and altitude), significantly influence the diversity indices of species (Nadal-Romero et al. 2014). CaCO3 and aspect showed negative relationships with vegetation indices. Study has demonstrated that slope and aspect are key limiting factors, exerting substantial influence over the spatial distribution of surface radiation (Bennie et al. 2008). This consequential influence subsequently affect patterns of evaporation dynamics and soil moisture content, which ultimately affects the species indices.

## Conclusions

In the present vegetation study, 252 species (39 trees, 43 shrubs, 167 herbs, 3 climbers) of 80 families were recorded in the study area. The overall results of SI showed that there was low similarity (below 50%) among the various communities in different stations. Due to the similar altitude and topography, S1 × S11 and S5 × S6 showed the highest SI value while S3 × S10 and S3 × S9 showed lower SI values due to large variation in their altitude and moisture contents. The results of the present study revealed that all diversity indices (species diversity, evenness index, species richness index, maturity index) during spring showed that the communities which were established in plains lying at lower altitudes (S1, S2, S6, S9, S10, S11) had higher values while the communities formed at high altitudes (S3, S4, S8) had lower values. As a whole, 12 communities were established in 12 different stations with different topographic and edaphic characteristics. The present study revealed that all diversity indices for communities in plains lying at lower altitudes had higher values. It can be concluded that variation in edaphic factors and topographic factors significantly affect the species distribution and diversity indices.

## Data Availability

This research work is part of the PhD thesis of the first author. Species occurrence data is available on request to first author.
